# Supplementary material for: Evidence supported by Mendelian randomization: impact on inflammatory factors in knee osteoarthritis
Source: Front Med (Lausanne). 2024 May 28;11:1382836. doi: 10.3389/fmed.2024.1382836 (PMC11165061; doi:10.3389/fmed.2024.1382836)
Supplement: Supplementary file 1 [file Data_Sheet_1.ZIP › data/GCST90274791_5e6/791.forest.pdf]

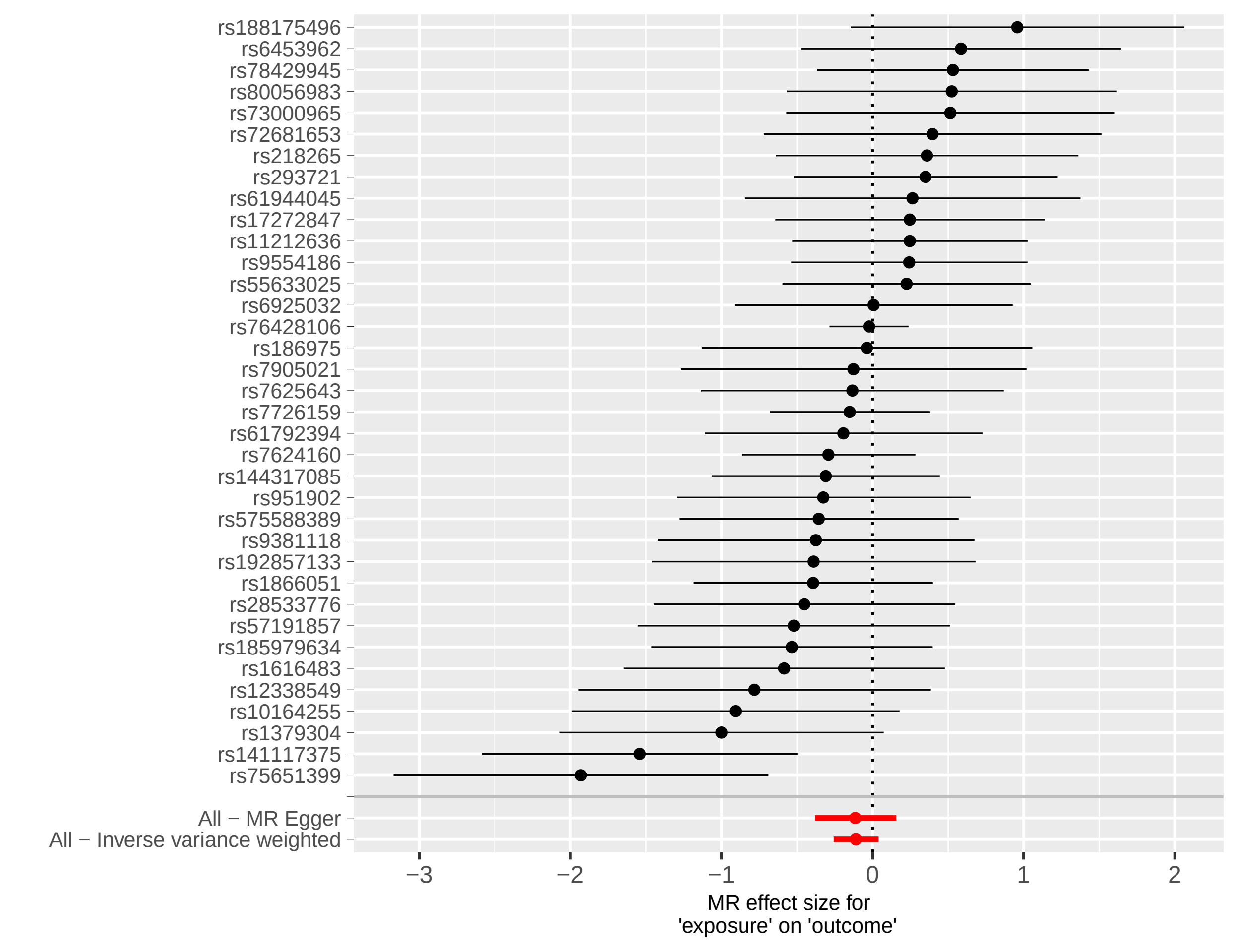
rs188175496
rs6453962
rs78429945
rs80056983
rs73000965
rs72681653
rs218265
rs293721
rs61944045
rs17272847
rs11212636
rs9554186
rs55633025
rs6925032
rs76428106
rs186975
rs7905021
rs7625643
rs7726159
rs61792394
rs7624160
rs144317085
rs951902
rs575588389
rs9381118
rs192857133
rs1866051
rs28533776
rs57191857
rs185979634
rs1616483
rs12338549
rs10164255
rs1379304
rs141117375
rs75651399
All − MR Egger
All − Inverse variance weighted
−3
−2
−1
0
1
2
MR effect size for
'exposure' on 'outcome'
